# Description of bacterial RNA transcripts detected in *Mycobacterium tuberculosis* – infected cells from peripheral human granulomas

**DOI:** 10.1080/21505594.2025.2547326

**Published:** 2025-08-16

**Authors:** Philip J. Moos, Allison F. Carey, Jacklyn Joseph, Stephanie Kialo, Joe Norrie, Julie M. Moyareke, Anthony Amof, Hans Nogua, Albebson L. Lim, Louis R. Barrows

**Affiliations:** a Department of Pharmacology and Toxicology, University of Utah, Salt Lake City, UT, USA; b Department of Pathology, University of Utah, Salt Lake City, UT, USA; c Coordinator of Pathology Services, Port Moresby General Hospital, Port Moresby Papua New Guinea; d Division of Pathology, School of Medicine and Health Sciences, University of Papua New Guinea and Central Public Health Laboratory, Papua New Guinea National Department of Health, PMGH, Boroko, Papua New Guinea; e Department of Medicinal Chemistry, University of Utah, Salt Lake City, UT, USA

**Keywords:** *Mycobacterium tuberculosis*, scRNA-seq, lymph node granuloma, bacterial transcripts

## Abstract

*Mycobacterium tuberculosis* (Mtb) remains a global human health threat. However, understanding effects of the microbe on cellular interactions in infected tissue has been hindered by inability to discriminate between infected versus un-infected cells. We included the H37Rv Mtb reference genome when assembling scRNA seq libraries from fine needle aspirate samples of peripheral nodal TB patients. Using the 10X Genomics Cell Ranger tool to align sequencing reads, we consistently detected bacterial small and large ribosomal subunit RNA sequences. We interpret Mtb reads associated with a cell’s UMI and transcriptome to indicate infection of that individual host cell. This provides a new window into the status of infected cells in the context of the bystander cells in the infected tissue. We investigated these Mtb transcripts to explore their clinical utility. The Mtb transcripts showed frequent sequence variation from the reference genome, with greater than 90% of the *rrs* or *rrl* reads from many clinical samples having at least one sequence difference. The highly conserved nature of the *rrs* and *rrl* gene sequences limited the ability to assign bacterial lineage based solely transcriptome analysis. However, rapid improvements in sequencing depth may soon allow transcriptome analysis of infecting microbes and improved certainty regarding their lineage, drug resistance, and virulence factors.

## Introduction

Mtb remains a global human health threat and a significant cause of human morbidity and mortality [1]. Drug-resistant Mtb is becoming more prevalent, and so rapid description of resistance factors is becoming increasingly important [2]. Next-gen sequencing has facilitated studies of bacterial genomes and uncovered pathogen variants associated with clinically relevant phenotypes such as antibiotic resistance [3]. However, these studies are primarily focused on bacteria cultured from patient tissues, and thus viable but non-cultivable bacteria are not assessed. This yields an incomplete assessment of bacteria related to the infection. We document here the capture of bacterial transcripts in libraries designed to amplify eukaryotic mRNA. These reads are often considered spurious or nuisance and until recently were rarely investigated [4–7]. To investigate the potential utility of these unexpectedly detected bacterial RNA sequences, we present here a description of *Mycobacterium tuberculosis* (Mtb) ribosomal RNA sequences detected in host human cells obtained from peripheral lymph node aspirates from patients infected with Mtb.

The Mtb transcripts identified using 10X Genomics single cell RNA sequencing (scRNA-seq) are described here. The initial experiments detected Mtb transcripts detected in THP-1 cell/H37Ra co-culture experiments [8–12], some of which exhibited significant sequence variation compared to the reference H37Ra (NC_009525.1 [13]) genome. The clinical samples that validated the cell culture experiment observations were fine needle aspirate samples from patients presenting at the Central Public Health Laboratory TB clinic (CPHL), Port Moresby General Hospital, Papua New Guinea (PNG) [14]. Using 10X Genomics scRNA-seq transcriptomics pipeline [15], as in the co-culture experiments, we consistently detected bacterial small and large ribosomal subunit RNA sequences (*rrs* and *rrl*, respectively) and other bacterial gene transcripts in the patient sample scRNA-seq libraries. We interpreted Mtb reads associated with a host cell’s unique molecular identifier (UMI) and transcriptome to indicate infection of that individual host cell. The frequent Mtb transcript sequence variation observed in the co-culture experiments presaged an even higher degree of transcript variation observed in the clinical samples. Greater than 90% of the *rrs* or *rrl* reads from many clinical samples had at least one sequence difference [16] compared to the H37Rv reference genome. The repeated, nonrandom nature of the sequence variations detected in Mtb *rrs* and *rrl* transcripts from multiple patients, suggests that, even though this appears to be a stochastic process, there is possibly some selective pressure that limits the types and locations of sequence variation generated either by the analytic process or allowed physiologically. We believe our data show the potential utility of this approach to assess infecting bacterial transcriptomes for drug resistance or virulence factors, which could inform more effective management strategies. Understanding host cell responses to infection within accessible involved tissues, such as peripheral lymph nodes, could also provide new insight into disease progression.

## Results

### Detection of bacterial transcripts in THP-1 cells

**Figure 1.**
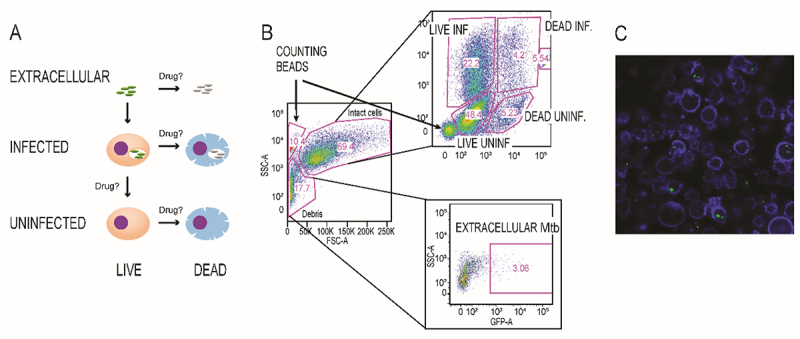
A) Cartoon showing THP-1 cell types quantified. THP-1 cells were differentiated with PMA for 24 h prior to infection and then co-incubated with GFP H37Ra for 5 days (MOI 2:1). B) Gating paradigm of intracellular and extracellular Mtb populations using forward and side scatter parameters. Internal standard counting beads quantify total cell count gain or loss. Intact cells were plotted versus V450 viability stain (abscissa) and GFP expression (ordinate). Flow cytometry conducted with a BD canto, results analyzed using FlowJo™ software. Extracellular GFP-Mtb were in the “Debris” gate. C) Confocal microscopy of THP-1 cells infected with GFP H37Ra confirmed intracellular Mtb. Cells were fixed in 1% formaldehyde. Actin was stained with Abnova™ fluorescent dye 405-I Phalloidin (blue). The image was acquired on a Nikon A1 confocal microscope using a 60x oil lens and processed using Fiji™ software. Image is an average intensity projection of 6 z-stacks, spaced 0.5 µm apart.

**Figure 2.**
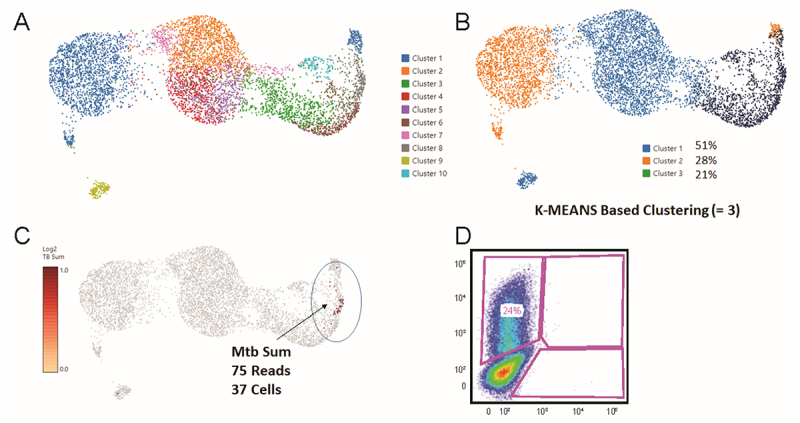
A) Loupe UMAP of THP-1/Mtb co-culture (14800X6) 5 days after inoculation of 0.5X10^6^ cells with GFP H37Ra. THP-1 cells were differentiated with PMA for 24 h prior to infection and then co-incubated with GFP H37Ra for 5 days (MOI 2:1). B) Same data at K means clustering, K = 3, yielded cluster 3, approximately equal to percent GFP expressing cells detected in replicate culture by flow cytometry. C) Feature plot showing any cell containing an Mtb transcript. All Mtb containing cells were found in cluster #3. D) Flow cytometry analysis of duplicate culture showing GFP fluorescence (percent infection) on vertical axis.

In order to more fully describe drug actions on the intracellular course of Mtb infection, we developed a flow cytometry-based system to quantify drug effects on different cellular compartments observable in THP-1/Mtb *in vitro* co-cultures, using GFP-expressing H37Ra (Figure 1) [17–23]. We confirmed that GFP expressing THP-1 cells contained intracellular Mtb using confocal microscopy (Figure 1). We also then conducted scRNA-seq analysis on parallel cultures to seek transcriptional signatures of infection that might serve as valuable identifiers of infected cells from clinical samples (Figure 2).

The co-culture system routinely yielded around 20% infection of THP-1 cells, defined by GFP expression 5 days after Mtb co-culture. Replicate cultures analyzed by scRNA-seq (Figure 2, Fig. S-1) were assessed for GFP expression using flow cytometry (Figure 2(D)) and showed from 15% to 24% infection for the replicate experiments, respectively. Each replicate experiment was processed with a technical repeat, e.g. samples 14,800 × 3 and -X4 and 14,800 × 5 and -X6. Unsupervised UMAP clustering of the THP-1/Mtb co-cultures at default 0.08 resolution did not yield clusters matching the percentages of GFP expressing cells determined by flow cytometry (Figure 2(A)). However, K-Means based clustering at K = 3 yielded cluster sizes almost exactly matching the respective percent GFP positive by flow cytometry for the given replicates (Figure 2(B), Fig. S-1). Early literature suggested the possible presence of polyadenylated transcripts in Mtb RNA [24], and so we included the Mtb H37Rv reference genome (NC_000962.3 [13]) in the Cell Ranger genome alignment and queried if any Mtb sequences were associated with THP-1 UMIs (xf:i:25.sam reads) and plotted them on the feature UMAP (Figure 2(C), Fig. S-1). Thus, single-cell RNA sequencing of duplicate cultures confirmed H37Ra *rrs* or *rrl* transcripts in GFP-expressing THP-1 cells. Flow cytometry parameters were set to count a minimum of 30,000 events. An average of about 30 infected (Mtb+) cells was detected in each experiment after QC, meaning that *rrs* or *rrl* sequences were only detected in about 4% of the GFP Mtb-infected cells. While the percentage of detected host THP-1 cells containing Mtb transcript sequences was low, all of these cells clustered in the presumed “infected clusters” determined by the percent GFP-positive cells in the parallel duplicate experiments (Figure 2(C), Fig. S-1).

**Figure 3.**
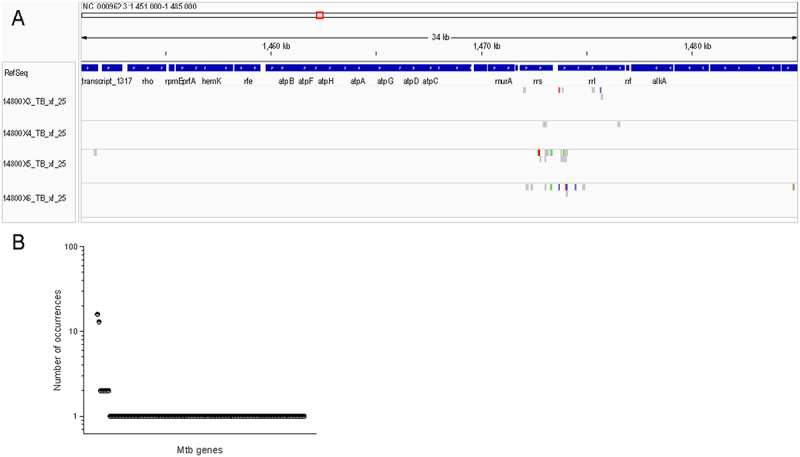
Integrated genome viewer alignment of reads detected in cell culture experiments with the H37Rv Mtb genome (NC_000962), centered on *rrs* and *rrl* genes. H37Rv and H37Ra (NC_009525) genomes are identical through the *rrs* and *rrl* genes. Two independent experiments, 14800X3&4 and 148,005&6, were conducted. Each experiment was completed with full technical repeats designated 3 & 4 and 5 & 6 (respectively). A) Shows all Mtb reads detected throughout the *rrs* and *rrl* genes for the repeat co-culture experiments. Sequence deviations from the reference genome are indicated by color bars; changes to G (brown), C (blue), a (green), and T (red). B) Reads of given Mtb genes detected co-culture experiments. Reads for *rrl* and *rrs* were detected most frequently. Reads for other genes with two copies detected were *Rv*0636, *Rv*1095, *Rv*1461, *Rv*1899*c*, *Rv*3616*c,* and *Rv*3803*c*. The remaining transcripts detected only once totaled 130 genes.

Comparison of the detected Mtb sequences in repeat experiments to the reference *rrs* and *rrl* sequences (synonyms *Rvnr*01 and *Rvnr*02, respectively) in the H37Rv and H37Ra genomes (Rv and Ra are identical through the *rrs* and *rrl* genes) showed that approximately 58% (17/29) of the reads from the co-culture experiments contained at least 1 sequence variation, almost 17% of the reads (5/29) contained multiple sequence variations (Figure 3(A)). These sequence variations were initially attributed to transcription errors in the 10X Genomics amplification process, but assessment of clinical samples, below, suggests that this is not a totally random occurrence and that additional factors may be contributing to the high read-sequence variability. The number of reads per gene across the Mtb genome was summed, and transcripts for *rrs* and *rrl* far outnumbered the other genes detected, likely because of high transcription rates of ribosomal RNA during infection. Most other gene transcripts were detected once, while a few were detected twice, *Rv*0636, *Rv*1095, *Rv*1461, *Rv*1899*c*, *Rv*3616*c*, and *Rv*3803*c* (Figure 3(B)). Interestingly, *Rv*3616*c* is a crucial virulence gene, and *Rv*3803*c* is a major antigen, and thus may also represent highly transcribed genes [25,26].

### Detection of high sequence variation in Mtb transcripts from clinical samples

**Figure 4.**
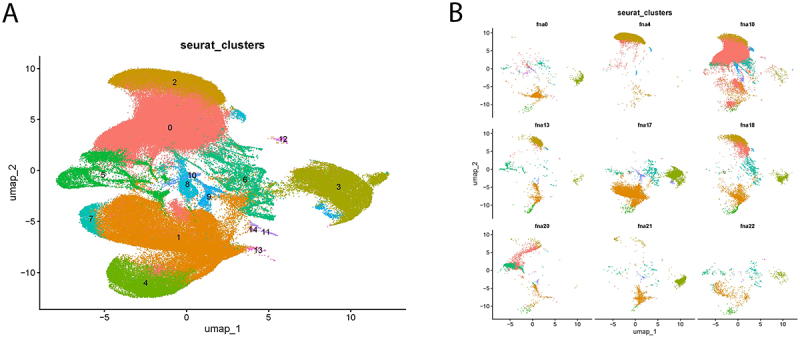
A) Annotated cluster identification of combined FNAs UMAP. B) UMAPs of the nine individual patient samples for which pathology lab confirmed the presence of Mtb in FNA samples.

Bacterial transcripts were detected in 21 of the 24 patient sample scRNA-seq libraries [14]. The combined data set UMAP of all 24 patients is shown in Figure 4(A). In several clinical samples, the transcript variation in the detected reads, combined with the highly conserved nature of bacterial *rrs* and *rrl* genes, actually made it impossible to confirm Mtb as the infecting organism solely based on sequence homology. Therefore, to provide confidence that the bacterial transcripts detected in the clinical samples actually arose from Mtb, the data presented here includes only bacterial sequences from nine individual patients with TB infection (Figure 4(B)) that were confirmed by the CPHL pathology lab using acid-fast microscopy and/or GeneXpert™ analysis [27]. While this does not exclude the possibility that other co-infecting bacteria could have been present in the patient’s granuloma in addition to Mtb, it does independently confirm that Mtb was present in each of these samples. This is a standard used previously in the assessment of nodal tuberculosis granulomas [28]. As in the co-culture experiments, above, bacterial *rrs* and/or *rrl* transcripts were most frequently detected. Over 90% of the transcripts in the *rrs* or *rrl* genes in most of the clinical samples contained at least one sequence difference, most of them showing multiple differences, when compared to the reference H37Rv genome. Similar sequence variation was also observed in the transcripts of other genes that were sporadically detected in the clinical samples. The *rrl* and *rrs* genes are highly conserved across the different Mtb lineages [29], and so it was determined that the sequence differences seen here were not due to differences between lineages.

**Figure 5.**
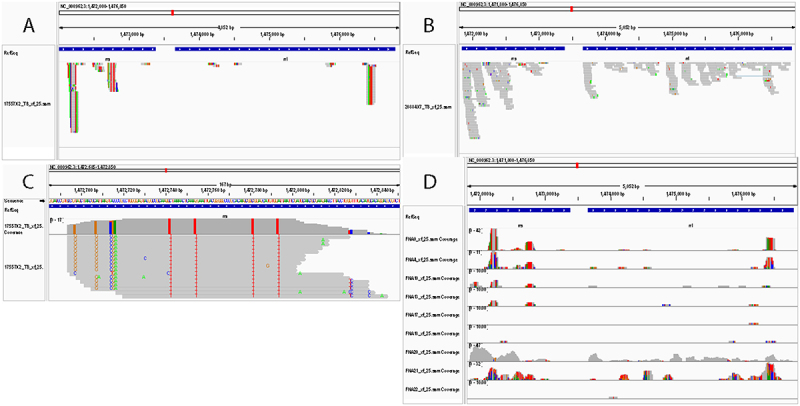
Repeated sequence deviations from the reference genome in Mtb reads. A, B) IGV alignment of intracellular Mtb reads from FNA 0 and FNA 20, respectively. Sequence variations versus the reference H37Rv genome are show in colors: G (brown), a (green), C (blue) and T (red). FNA 0 had a high incidence of repeated sequence variations, FNA 20 had a relative low incidence of sequence variations. C) A close up view of a stack of reads from FNA 0 shows sporadic sequence variations observable in independent reads (attributed to random errors in the scRNA seq pipeline). Multiple reads of identical length, starting position and directionality, usually in pairs or sets of three, are interpreted as arising from a single RNA source which we hypothesize was then amplified during library generation. Repeated/retained sequence variations are also detectable, indicated by their repetition in multiple stacked reads in this view. D) All intracellular Mtb reads from the nine Mtb-confirmed FNA samples presented here in a coverage plot. Even though the samples were gathered from nine different individuals, one in 2019, one in 2022 and seven in 2023, the sites and types of sequence variation are repeated among individual infections, suggesting that the process is not random and is possibly constrained to “hard to map” gene regions or by the functionality of the changed sequence. Patterns of variation overlap and repeat in patient samples.

Figure 5 shows contrasting examples of Integrative Genomics Viewer (IGV) alignments of Mtb reads from two patient samples with relatively high levels of bacteria transcripts compared to the other FNAs analyzed (Figure 5(A,B)). FNA IGV stacks of coverage from all reads from the nine FNAs are presented in Figure 5(D). The frequency of detection of bacterial reads was not consistent across all patient samples. The detection of Mtb reads was low in samples FNA 17, FNA 18, and FNA 22. Whereas in FNA 0, FNA 4, FNA 10, FNA 13, FNA 20, and FNA 21 the number of detected Mtb reads was relatively high, and higher than that observed in the co-culture experiments. The degree of sequence variation also differed significantly among the patient samples. For instance, FNA 0 displayed higher frequencies of sequence variations from the reference H37Rv genome than FNA 20, which showed very few relative to the other samples analyzed. FNA 0, FNA 4, FNA 10, FNA 13, and FNA 21 contained higher numbers of reads exhibiting repeated nucleotide variations, compared to FNA 20 and FNA 22, with FNA 10 showing an intermediate frequency of variation.

Closer inspection of the transcript variations revealed several details about the detected reads. First, many single nucleotide variations were repeated in multiple reads, thus appearing to be a retained feature rather than a random event. We hypothesize that identical length and directionality of the xf:i:25 reads indicated that the reads were likely replicated amplification products of the 10X Genomics pipeline (Figure 5(C)). Reads of identical length almost always exhibit the same commonly repeated sequence variations. We attribute the random single nucleotide variations in individual FASTQ xf:i:25 reads sequences of identical length to stochastic errors introduced by the 10X Genomics sequencing process. However, if this interpretation is correct, then the repeated variations detected over multiple transcripts (reads of different lengths and of opposite reading orientations) would seem to suggest that the variation arises from a repeated error-prone process during library generation, or alternatively, it could also indicate some selection pressure during transcription, resulting in only certain variations.

**Figure 6.**
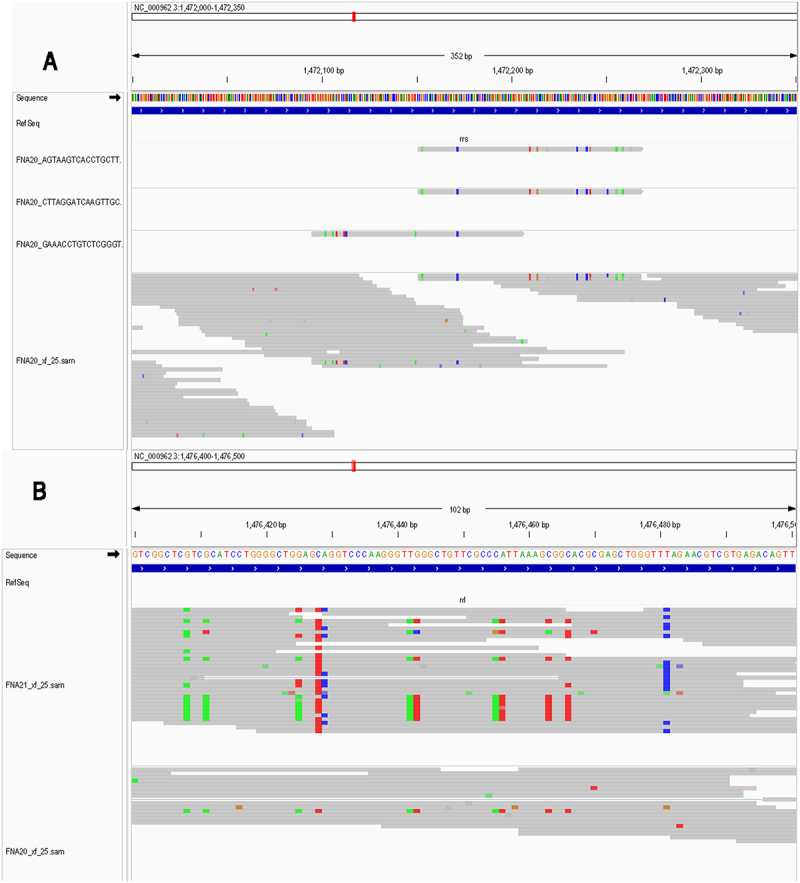
A) IGV view of intracellular reads from FNA 20 associated with different host cell UMIs. Overlapping reads from different host cells, and thus different infecting bacteria, retain/repeat nucleotide polymorphisms (gray bars are lower quality sequence calls). B) Overlapping Mtb transcript reads from two different patient samples also show similar patterns of sequence deviations from reference genome.

Mtb transcript sequences that are associated with different UMIs clearly represent different source bacteria, although those from the same FNA might be attributed to a single inoculum. Nucleotide variation is conserved in overlapping RNA reads from bacteria associated with different UMIs (Figure 6(A)). The repetition of similar Mtb transcript variations associated with different UMIs shows that some transcript nucleotide variations occur repeatedly, even in different host cells. Comparison of overlapping reads from two different patient samples also shows repeated transcript sequence variations (Figure 6(B)). This indicates that the repeated variants do not arise from a single inoculum of a given patient.

### Some reads are confirmed to be Mtb by BLAST analysis

When general BLAST (blastn [16]) searches were conducted with the FASTQ *rrs* and *rrl* sequences from different patients, Mtb was the top match in only three of the nine samples presented here (FNA 10, FNA 20, and FNA 22), even though all nine FNAs were confirmed as Mtb positive by acid fast microscopy or GeneXpert® analysis.

In further efforts to utilize the detected reads to confirm bacterial identity, additional BLASTn searches were conducted. For FNAs with transcripts detected other than *rrs* and *rrl* (FNA 10 had six other genes detected and FNA 20 had 573 other genes detected), BLASTn searches of those gene transcript sequences matched only mycobacteria, >90% match to Mtb with and occasional match to *M. bovis* and rarely to *M. canettii* or *M. microti*. No non-mycobacteria were matched. Even at the lowest alignment identity (80%) for some of the FNA 20 gene transcripts, the only matching organism was *M. tuberculosis*. BLASTn searches were conducted for all FNAs where transcripts below the normal NGS error rate were detectable (FNA 0, 4, 10, 20, 21, and 22). For FNA 0, 20, and 22 these sequences matched only Mtb when the number of mismatches was also restrained to 0 or 1; however, when the number of mismatches was higher (for example >3 in FNA 20) then “uncultured bacteria” and other bacteria could align with higher scores than Mtb. For FNAs 0 and 4, the best alignments were with “uncultured bacteria.” For FNA 21 the top alignment was *Corynebacterium pseudotuberculosis*, also a possible intracellular pathogen (although not acid-fast). For all BLASTn searches of bacterial transcripts from the THP-1 & H37RA co-culture experiments, the only matches were to mycobacteria.

In an attempt to assign our FASTQ reads to a known Mtb lineage, and because H37Rv is a laboratory-adapted reference strain, and does not belong to the Mtb lineage predominant in PNG [30], we compared *rrs* and *rrl* sequences from the published WGS genomes in the reference set of clinical samples recommended by Borrell et al. [29]. Overall, there is very high identity between the published WGS data sets for these two genes across all lineages, and it was not possible to assign lineage using the respective published *rrs* and *rrl* sequences. Thus, we found it was not possible to use the bacterial transcripts of *rrs* or *rrl* detected by the 10X Genomics scRNA-seq pipeline to assign lineage identity to our infecting Mtb. This was disappointing, because we found the sensitivity of this pipeline for the detection of bacterial reads rivals PCR. Several PCR attempts were made to detect transcripts of *Rv*1467*c*, *RRDR*, and other Mtb genes in our source re-hydrated cells, with no success.

### Detection of non-ribosomal Mtb transcripts in clinical samples

**Figure 7.**
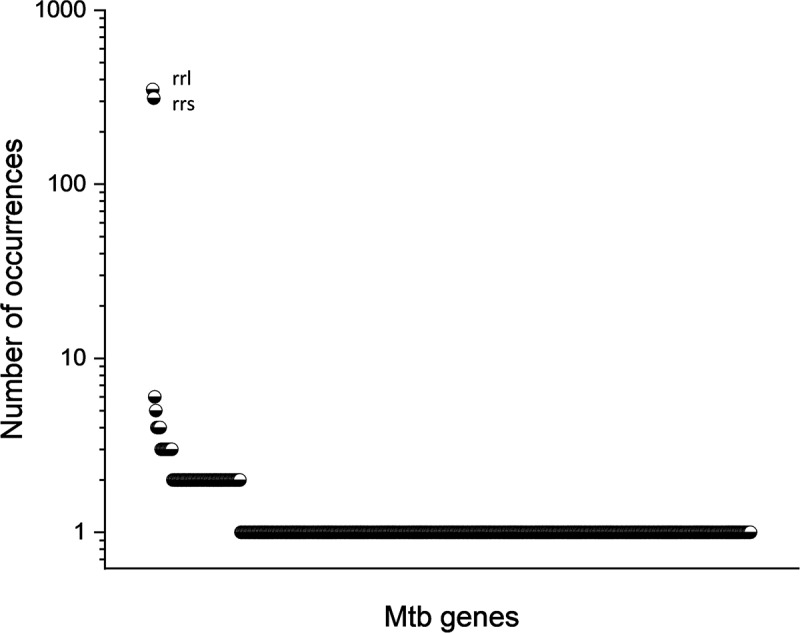
Distribution of detected Mtb RNAs throughout host cells of combined FNA samples. Plot showing frequency of Mtb transcript detection in combined Mtb positive FNAs. Transcripts for *rrl* and *rrs* were detected in 342 and 304 cells, respectively. Other gene transcripts detected included TB-*Rv*2186*c* that was detected in six cells. A total of 564 different Mtb gene transcripts were detected in the combined data set.

*Rrs* and *rrl* were the most frequently detected Mtb transcripts in the clinical samples (Figure 7), with *rrl* and *rrs* being detected in 342 and 304 cells, respectively. However, these transcripts were not detected in all host cells. Other gene transcripts detected included TB-*Rv*2186*c* that was detected in six cells. The transcript for TB-*Rv*2553*c* was detected in five cells; TB-*Rv*3343*c*, TB-*Rv*2490*c*, TB-*Rv*2319*c* andTB-*Rv*0895 were each detected in four cells. Eleven other Mtb transcripts were each detected in three individual cells, 84 different Mtb gene transcripts were detected in two cells, and 480 other Mtb gene transcripts were detected in only one cell. A total of 564 different Mtb gene transcripts were detected in the combined data set. In the future, improving coverage of scRNA-seq pipelines may soon provide sufficient lineage discriminating non-rRNA reads for assignment of lineage.

### Detection of rrs or rrl mutations associated with drug resistance in clinical samples

FNA 20 had sufficient coverage of *rrs* and *rrl* transcripts to ask if any of the detected variations would coincide with known drug resistance markers. When compared against the catalog of drug resistance mutations in Mtb published by WHO [31], no transcript reads displaying such mutations were detected.

## Discussion

We present here the description of Mtb RNA sequences detected by single cell transcriptomic analysis of fine needle nodal granuloma aspirate samples from TB patients. Even though the 10X Genomics single-cell RNA sequencing pipeline [15] is designed to capture eukaryotic mRNA, bacterial RNA sequences were detected in all nine of the clinical samples confirmed for Mtb infection. Cell culture experiments using THP-1 cells and GFP-expressing Mtb showed that the detection of Mtb *rrs* and *rrl* transcripts associated with host cell UMIs was reliable but low efficiency, estimated at approximately 4%. In scRNA-seq analysis of 12 control, bacteria-free, THP-1 cell cultures (each conducted with a technical repeat), only one read was ever detected that identified as an Mtb sequence, showing the remarkable selectivity of this analytical tool. We attribute that sole outlier to a sequencing errors inherent to 10X Genomics pipeline, it did not align to *rrs* or *rrl* and it did not occur in its technical repeat. The ability to detect individually infected cells, cluster them, and compare their transcriptomes to uninfected cells in this co-culture system suggested the possibility of performing similar analysis on tuberculosis patient granuloma samples and provided the impetus for the translational studies reported here. Such analyses have the potential to identify circulating Mtb strains, drug sensitivities, and virulence characteristics as well as providing insight into cellular interactions and functions of infected cells versus uninfected cells within nodal granulomas [14].

BLAST analysis of the Mtb reads detected in the cell co-culture system uniformly identified Mtb as the top match in every case. This certainty was obtained in only three of the nine clinical samples discussed here. Nevertheless, we believe that the bacterial transcripts detected in the six clinical samples that did not identify Mtb as the top match using BLASTn, are likely also Mtb in origin for several reasons. First, Mtb infection was confirmed in all these patient samples using acid fast microscopy or GeneXpert analysis. There were no clinical signs of co-infecting organisms identified in the sampled patients. Second, all the detected sequences were associated with host cell transcripts indicating intracellular infection, characteristic of Mtb infection. Third, the sequence variations identified in this study, by definition, decrease the sequence homology to the reference genome decreasing the identity of match to the reference H37Rv genome. Fourth, the sequence variations themselves appeared to be a continuum ranging from low to high frequency among the patient samples. And, finally, the characteristic repeated sequence variations identified in the infecting bacterial *rrs* and *rrl* transcripts, are repeated among many of the patient samples, differing primarily in the frequency with which they are detected. Those samples in which the sequence variations in the detected FASTQ reads were fewest were the ones that matched Mtb best, using BLASTn, yet they still exhibited some of the same sequence variations repeated in the other six samples. Nevertheless, it is important to acknowledge the possibility that the granuloma FNAs may harbor other infecting bacteria, in addition to the Mtb, and that the bacterial reads we are analyzing may not arise solely from Mtb, but could represent a mix of multiple infecting bacteria. Thus, although Mtb was confirmed by clinical methods (GeneXpert, AFB), transcript-level taxonomic resolution is limited due to short reads and high conservation of rRNA genes.

Deviation of detected RNA sequences from the reference genome is to be expected. The default cutoff we used in the Cell Ranger transcriptome assembly was 10 mismatches per read (approximately 150 nucleotides). This accommodates the error-prone processes of library generation and sequencing. Indeed, these expected random error single nucleotide substitutions were detectable in most individual transcript reads. Approximately 58% of the reads from the co-culture experiments contained at least one nucleotide substitution, approximately 17% of the reads contained multiple sequence variations. Even higher rates of transcript sequence variation were observed in the clinical samples. Over 90% of the detected reads in most of the clinical samples contained at least one sequence variation and many reads contained multiple variations, and uniquely, most of these variations were repeated in overlapping reads. We do not believe the RNA sequence variations reflect bacterial DNA mutation because of the consistency in the published Mtb lineage WGS sequences for *rrs* and *rrl* [29]. Those WGS sequences are often derived from sub-cloned bacteria cultured from sputum samples, and thus would be expected to show diversity similar to our detected RNA reads if the RNA read sequence diversity arose from the genome level.

The number of detected Mtb reads differed significantly among the patient samples. Detection of bacterial reads was scant in samples FNA 17, FNA 18, and FNA 22. Whereas some samples, such as FNA 0, FNA 4, FNA 10, FNA 13, FNA 20, and FNA 21 exhibited high levels of detected bacterial transcripts. We speculate that levels of detected bacterial transcripts might reflect the relative bacterial load in these patients and are attempting to confirm this in on-going experiments. If that is the case, then we might be able to stratify our patient samples into high bacillary versus low bacillary load categories, which would be informative for analyzing which immune parameters contribute to Mtb control in granulomas [32]. The degree of the nucleotide sequence variation also differed significantly among the patient samples. Some samples, like FNA 20 exhibited fewer variations per read, and other such as FNA 0, FNA 4, FNA 10, FNA 13, and FNA 21 exhibited higher degrees of variation.

We do not know how or why the 10X Genomics pipeline seems to detect bacterial rRNA so readily. One reason could be the high degree of transcription of these genes. A second possibility is that the secondary structure of the *rrs* and *rrl* RNAs can self-prime replication or are particularly susceptible to priming by the UMI or barcode regions of the poly-T primers. To explain the sequence variations, it is possible that the modified nucleotides or complex secondary structures that are common in rRNA, miscode occasionally when replicated and amplified in the 10X Genomics pipeline. “Hard to map regions” and contaminant DNA are known causes of sequence variation in DNA sequencing [33], and similar problems might apply here.

The sequence variations in the bacterial *rrs* and *rrl* transcripts were not random, but rather appeared to be reproduced transcription nucleotide sequence variations common to multiple infecting bacteria. Many of the sequence changes appeared in multiple transcripts, originating from multiple individual bacteria, and in bacteria from different patients obtained years apart. If one accepts the possibility that the repeated sequence variations seen in the bacterial transcripts actually reflect the transcript sequence, then the data imply that some selective pressure results in preferred sequence alterations in certain regions of the *rrs* and *rrl* RNAs.

In conclusion, we have attempted to investigate the possible utility of bacterial RNA transcripts detected in human scRNA-seq libraries. We show that scRNA-seq analysis of nodal human tuberculosis FNA samples is achievable in a resource-limited setting, not requiring refrigerated centrifuges, culture hoods, etc. We also have shown that, unintendedly, scRNA-seq analysis captures RNA from infecting Mtb and can identify the individual cells that harbor the intracellular pathogen [15]. It was found that, in this current set of samples, sequencing coverage of the detected transcripts was insufficient to identify the lineage, drug resistance, or virulence markers of the infecting Mtb. However, the efficiency and depth of coverage obtainable with single cell RNA analysis is improving and may soon achieve this goal via analysis of more lineage-differentiating transcripts than *rrs* and *rrl*. It was encouraging to demonstrate that for one of the FNA samples (FNA 20) there was sufficient coverage of *rrs* and *rrl* genes to identify possible mutations of ribosomal RNA associated with drug resistance, suggesting the possibility that this approach could one day be used for diagnostic purposes.

## Materials and methods

### Bacterial culture

For Mtb *gfp*, bacteria were initially grown on 7H10 supplemented with OADC and kanamycin (KAN; 50 μg/mL). A starter culture of around 10 mL from an isolated colony was grown in 7H9 supplemented 10% ADC, 0.2% glycerol (v/v), 0.05% tween 80 (v/v), and KAN (50 μg/mL) at 37°C until it reached mid-to-late log phase, measured by OD_600_. An aliquot of the starter culture was added to ~100 mL of 7H9 with KAN (50 μg/mL) and grown to log phase, as monitored by OD_600_. GFP H37Ra was a gift from Prasit Palittapongarnpim, Department of Microbiology, Mahidol University, Thailand [17,18]. Middlebrook 7H9 broth and OADC medium supplement were obtained from BD Biosciences (cat. # 271310 and 211,886, respectively; San Jose, CA). Middlebrook 7H10 agar and ADC were obtained from Remel (cat. # R453982 and 705,565, respectively; Lenexa, KS). Glycerol was obtained from Acros Organics (cat. # 41098–5000; Fair Lawn, NJ) and Tween 80 from MP Biomedicals, Inc. (cat. # 103170; Santa Ana, CA).

### Cell culture

THP-1 cells were grown in RPMI 1640 with 10% FBS and 50 μg/mL KAN in 5% CO_2_ at 37°C. THP-1 cells were obtained from ATCC (Cat#TIB-202). HyClone™ RPMI 1640, kanamycin sulfate, Corning™ Accutase™ detachment solution and phorbol 12-myristate 13-acetate (PMA) were obtained from Fisher Scientific (cat. # SH30011.03, BP906–5, MT25058CI, and BP685–1, respectively). Fetal bovine serum was purchased from Atlanta Biologicals (cat. # S11150). BD Horizon™ Fixable Viability Stain 450 was obtained from BD Biosciences (cat. # 562241). Fluorescent Dye 405-I Phalloidin was purchase from Abnova™ (cat. # U0278).

### GFP Mtb-THP-1 cell co-culture

THP-1 cells were pre-incubated overnight in PMA (20 ng/mL) at 500,000 cells/well in order to generate differentiated macrophages. Medium was replaced for THP-1 cells 1-h prior to the addition of GFP-Mtb. Preliminary bacterial concentration experiments were performed in THP-1 cells prior to co-culture experiments. An MOI of 2:1 was chosen due to its ability to achieve a high degree of Mtb infection (~20%) with minimal toxicity to the cells following 24-h co-incubation. Mtb was added to THP-1 cells and incubated for or Mtb 5 days followed by preparation for analysis by flow cytometry and cell imaging or scRNA-seq analysis.

### Flow cytometry

Flow cytometry was performed as previously described [34]. “Adherent THP-1 cells were incubated with Accutase™ for 15 min at 37°C. THP-1 cells were transferred to 5-mL tubes and washed with PBS. Cells were resuspended in BD Horizon™ Fixable Viability Stain 450 (0.25 μg/mL) and incubated at 4°C for 30 min. Cells were then fixed in 2% formaldehyde for 30 min at 4°C. Cells were analyzed using a FACS Canto. Percent infection by DHIV was quantified as a subset of the live population (FSC/V450/50-). Gates for infection were set according to the uninfected ‘mock’ THP-1 cell controls. Three independent biological replicates were completed for all treatment conditions, each in triplicate wells per experiment. Population analysis was then done using FlowJoTM v10.7 [35], to assess if infection levels and cell viability were consistent similar in all replicates. The Flow Cytometry figures are representative plots obtained from one of the replicates.” A minimum of 30,000 events were collected per experiment.

### Cell imaging

Following Accutase™ removal of adherent THP-1 cells, THP-1 cells were washed with PBS fixed with 2% formaldehyde at 4°C for 30 min. Cells were resuspended in 100 μL of a 1:1000 dilution of Fluorescent Dye 405-I Phalloidin in PBS and incubated for 15 min at room temperature. The images were acquired on a Nikon A1 confocal microscope using a 60x oil lens. Images were processed using Fiji [36].

### Performance of experiments in accordance with relevant guidelines and regulations statement

Medical Review Council (Institutional Review Board equivalent) approval for the work described here was obtained by Dr.s Evelyn Lavu (decd), Rodney Itaki, and Louis R. Barrows from UPNG School of Medicine and PNG Science and Technology Secretariat. All experiments were conducted in adherence to the Declaration of Helsinki. Current experiments performed under the aegis of Dr. Jacklyn Joseph, Coordinator of Pathology Services, Port Moresby General Hospital. All experiments were performed after obtaining verbal patient prior informed consent. Verbal consent was obtained due to the patients often being illiterate in English. All experiments were done in accordance with relevant guidelines and regulations. Approved protocols allowed up to 3 passed of fine needle aspiration using a 22-gauge needle for sampling lymph nodes of 0.8 cm diameter or larger.

### Preservation of fine needle aspirates for genomic and transcriptomic analysis

Under the University of Papua New Guinea School of Medicine-Medical Research Council approved protocol, patients presenting at the CPHL TB Clinic, and tentatively diagnosed with LNTB, upon giving informed consent, were subjected to the standard diagnostic protocol, which includes FNA of enlarged (>1.0 cm) lymph nodes. This aspirate goes for microscopy and for GeneXpert analysis as part of the patient assessment process. Aspirate from one pass of the granuloma dedicated to this study was washed directly into ice-cold RPMI buffer containing 0.2% fetal bovine serum, in a heparinized tube. The samples were taken to the adjacent pathology lab where they are pelleted for 5 min. and suspended on ice in NH_4_Cl lysis solution [37] to lyse contaminating erythrocytes. After a maximum of 5 min with occasional gentle mixing on ice, and observation of the depletion of obvious erythrocytes, 1 mL of Accutase™ was added directly to the lysis buffer for a maximum of an additional 3 min., again with occasional gentle mixing and observation of the dissolution of obvious tissue clots in the solution. The sample volume was expanded with ice cold RPMI buffer and the cells were pelleted again. The pelleted cells were gently suspended in 200 µl preservation buffer to which 1 mL of ice-cold methanol is slowly added with mixing. The de-identified samples are kept on ice packs for transportation and analysis by PCR, WGS, and scRNA-seq.

### Single-cell RNA-sequencing

scRNA-seq was performed on single-cell suspensions using the 10X Genomics Chromium protocol to prepare cDNA sequencing libraries as described by Brady et al. [37]. Samples were processed using the Chromium Single Cell 3′ V3 Kit (10X Genomics, Cat. # 10,00075) using whole cells fixed in 80% methanol. Single cells were diluted to a target of 1000 cell/μL in 1× PBS (whole cells) or 1× PBS + 1.0% BSA +0.2 U/μL RiboLock RNase Inhibitor to generate GEM’s prepared at a target of 10000 cells per sample. Barcoding, reverse transcription, and library preparation were performed according to manufacturer instructions. 10X Genomics generated cDNA libraries were sequenced on Illumina HiSeq 2500 or NovaSeq 6000 instruments using 150 cycle paired-end sequencing at a depth of 10K reads per cell. scRNA-seq was performed at the High Throughput Genomics Core at Huntsman Cancer Institute (HCI) of the University of Utah.

For data analysis, the 10X Genomics Cell Ranger Single Cell software pipeline [15] was deployed to produce alignments and counts, utilizing the prescribed default parameters. The genomic references used for alignment were the human (hg38), the H37Rv Mtb (NC_00096.3:1) and HIV-1 (NC_001802.1). For quality management and further analytical exploration, Seurat (4.1.0) was utilized. Doublets were identified with DoubletFinder. Cells were excluded based on having less than 100 genes/features and an excess of 25% mitochondrial genes. Mitochondrial genes were filtered out but every cell that contained Mtb genes was retained. Dimensionality was reduced and scaled via SCTransformation (0.3.5) using the Gamma-Poisson generalized linear model (glmGamPoi, 1.4.0) methodology at default resolution or less. Automated categorization of cells was performed using SingleR (1.6.1) [38–51]. Statistics within Seurat pipelines were generated with FindAllMarkers or FindMarkers which utilizes a Wilcoxon rank sum test [52].

## Data Availability

Sequence data that support the findings of this study have been deposited in the NCBI Gene Expression Omnibus; accession numbers: GSE281148 (https://www.ncbi.nlm.nih.gov/geo/query/acc.cgi?acc=GSE281148) and GSE281835 (https://www.ncbi.nlm.nih.gov/geo/query/acc.cgi?acc=GSE281835). Supplementary figures can be found in figshare: 10.6084/m9.figshare.28477253. Quality assessment of basic.bam data is provided in supplemental data: 10.6084/m9.figshare.29386652.

## Statistical analysis

Unpaired t tests were used to determine statistical significance across infection culture conditions of THP-1 from three independent experiments. Significance was determined and the level recorded if the p-value was less than 0.05.

The pairwise TTests function from Scran was used to determine statistically significant differential expression of genes between groups. This was performed for all comparison sets. Other default statistical standards were adopted from the various software recommendations during data analyses unless otherwise specified.
